# Aldosterone Jeopardizes Myocardial Insulin and β-Adrenergic Receptor Signaling *via* G Protein-Coupled Receptor Kinase 2

**DOI:** 10.3389/fphar.2019.00888

**Published:** 2019-08-09

**Authors:** Alessandro Cannavo, Federica Marzano, Andrea Elia, Daniela Liccardo, Leonardo Bencivenga, Giuseppina Gambino, Claudia Perna, Antonio Rapacciuolo, Antonio Cittadini, Nicola Ferrara, Nazareno Paolocci, Walter J. Koch, Giuseppe Rengo

**Affiliations:** ^1^Department of Translational Medical Sciences, Federico II University of Naples, Naples, Italy; ^2^Center for Translational Medicine, Temple University, Philadelphia, PA, United States; ^3^Department of Advanced Biomedical Sciences, Federico II University of Naples, Naples, Italy; ^4^Department of Cardiology, Istituti Clinici Scientifici ICS Maugeri S.p.A. IRCCS Istituto Scientifico di Telese Terme, Benevento, Italy; ^5^Department of Biomedical Sciences, University of Padova, Padova, Italy; ^6^Department of Cardiology, Johns Hopkins University, Baltimore, MD, United States

**Keywords:** aldosterone, mineralocorticoid receptor, GRK2, insulin, β-adrenergic receptor, heart failure

## Abstract

Hyperaldosteronism alters cardiac function, inducing adverse left ventricle (LV) remodeling either *via* increased fibrosis deposition, mitochondrial dysfunction, or both. These harmful effects are due, at least in part, to the activation of the G protein-coupled receptor kinase 2 (GRK2). In this context, we have previously reported that this kinase dysregulates both β-adrenergic receptor (βAR) and insulin (Ins) signaling. Yet, whether aldosterone modulates cardiac Ins sensitivity and βAR function remains untested. Nor is it clear whether GRK2 has a role in this modulation, downstream of aldosterone. Here, we show *in vitro*, in 3T3 cells, that aldosterone impaired insulin signaling, increasing the negative phosphorylation of insulin receptor substrate 1 (^ser307^pIRS1) and reducing the activity of Akt. Similarly, aldosterone prevented the activation of extracellular signal-regulated kinase (ERK) and the production of cyclic adenosine 3′,5′-monophosphate (cAMP) in response to the β_1_/β_2_AR agonist, isoproterenol. Of note, all of these effects were sizably reduced in the presence of GRK2-inhibitor CMPD101. Next, in wild-type (WT) mice undergoing chronic infusion of aldosterone, we observed a marked GRK2 upregulation that was paralleled by a substantial β1AR downregulation and augmented ^ser307^pIRS1 levels. Importantly, in keeping with the current *in vitro* data, we found that aldosterone effects were wholly abolished in cardiac-specific GRK2-knockout mice. Finally, in WT mice that underwent 4-week myocardial infarction (MI), we observed a substantial deterioration of cardiac function and increased LV dilation and fibrosis deposition. At the molecular level, these effects were associated with a significant upregulation of cardiac GRK2 protein expression, along with a marked β1AR downregulation and increased ^ser307^pIRS1 levels. Treating MI mice with spironolactone prevented adverse aldosterone effects, blocking GRK2 upregulation, and thus leading to a marked reduction in cardiac ^ser307^pIRS1 levels while rescuing β1AR expression. Our study reveals that GRK2 activity is a critical player downstream of the aldosterone signaling pathway; therefore, inhibiting this kinase is an attractive strategy to prevent the cardiac structural disarray and dysfunction that accompany any clinical condition accompanied by hyperaldosteronism.

## Introduction

Aldosterone is a corticosteroid hormone synthesized by the adrenal cortex mainly in response to renin–angiotensin system (RAS) activation and by high potassium (K^+^) levels (Hu et al., 2012; Cannavo et al., 2018a). Importantly, aldosterone activities were initially perceived as confined to a few organs, especially to the distal tubule and collecting duct of the nephrons in the kidney (Spät and Hunyady, 2004; Marney and Brown, 2007). However, the demonstration of specific aldosterone binding to its cytoplasmic/nuclear mineralocorticoid receptor (MR) in disparate areas of the cardiovascular system has widened the range of aldosterone targets (Marney and Brown, 2007; Schrier et al., 2010; Dooley et al., 2012; Verhovez et al., 2012). It is now consolidated, for instance, that this hormone controls the function and the growth, as well as the metabolism of several cell types, including cardiomyocytes, fibroblasts, and vascular cells (Brilla et al., 1993; Stockand and Meszaros, 2003; Hitomi et al., 2007; Cannavo et al., 2016; Cannavo et al., 2018a). This evidence has advanced aldosterone and its receptors to a new frontline for treating heart failure (HF), i.e., to prevent its onset or to arrest its progression (Brilla et al., 1993; Stockand and Meszaros, 2003; Hitomi et al., 2007; Cannavo et al., 2016; Cannavo et al., 2018a). Consistent with this new scenario, high aldosterone levels, and the subsequent MR hyperactivation, have been associated to the onset of insulin resistance, which is a well-recognized risk factor of cardiovascular disease (CVD) (Hitomi et al., 2007; Wada et al., 2009). More in detail, in vascular smooth muscle cells (VSMCs) and in adipocytes, aldosterone has been shown to inhibit insulin receptor substrate-1 (IRS1) that is considered a second messenger of the insulin receptor, thus preventing Akt activation and glucose uptake (Hitomi et al., 2007; Wada et al., 2009). Moreover, recent studies have demonstrated that, in fibroblasts, MR inhibition is able to prevent pro-fibrotic βAR activation (Reddy et al., 2015; Hori et al., 2017) and, as previously seen in the heart, aldosterone can both augment the hypertrophic response and myocardial apoptosis and fibrosis (Cannavo et al., 2016; Cannavo et al., 2018a). In this context, we have recently reported that, downstream of the aldosterone/MR system, there is the activation of G protein-coupled receptor kinase 2 (GRK2) and GRK5 (Cannavo et al., 2016). Accordingly, both *in vitro* and *in vivo*, we have found that aldosterone stimulation induces cardiac GRK2 activation, followed by increased cell death and mitochondrial dysfunction. Conversely, aldosterone-mediated GRK5 activation results in an increased pathological hypertrophy (Cannavo et al., 2016). Of note, GRK2 has been discovered as a regulator of cardiac contractility *via* β-adrenergic receptor (βAR) phosphorylation and subsequent desensitization in response to catecholamine stimulation (Cannavo et al., 2018b). However, in HF, a condition characterized by increased sympathetic nervous system activation (SNS), GRK2 activity/expression is upregulated, and this elevation in GRK2 activity has many adverse effects in the cardiovascular system because it induces massive βAR downregulation, with consequent left ventricular (LV) dysfunction (Cannavo et al., 2018b). Intriguingly, in addition to these canonical effects, GRK2 inhibits also IRS1, *via* direct binding and subsequent phosphorylation at the serine in position 307 thus leading to an impaired Akt activity (Garcia-Guerra et al., 2010; Ciccarelli et al., 2011; Mayor et al., 2011). Despite all this evidence, it remains to be determined still if aldosterone influences cardiac insulin signaling and βAR function and if this eventual modulation requires GRK2. Hence, in the present study, we tested the impact of aldosterone stimulation, and the role of GRK2, on both insulin and βAR signaling *in vitro*, in fibroblasts (3T3 cells), and *in vivo*, in mice undergoing chronic aldosterone infusion or surgical-induced myocardial infarction (MI), two models of hyperaldosteronism (Cannavo et al., 2016).

## Materials and Methods

### Agonists and Inhibitors

Aldosterone (A9477), spironolactone (S3378), insulin (I2643), and isoproterenol (ISO) (I5627) were purchased from Sigma-Aldrich (St. Louis, MO, USA). CMPD101 (HB2840) was purchased by Hello Bio (Bristol, UK).

### Cell Culture and Stimulation Conditions

3T3-L1 fibroblasts were purchased from ATCC (ATCC^®^ CL-173^™^) and cultured in Dulbecco's Modified Eagle Medium (DMEM) supplemented with 10% fetal bovine serum (FBS) and 1% P/S. Prior to stimulation, all the cells were starved with serum-free media. A group of cells was serum-starved for 12 h and then was stimulated with aldosterone (1 µM) at different time points in fresh serum-free media. Another group of cells was serum-starved for 1 h and then, under this condition, was pretreated with aldosterone (1 µM) and/or CMPD101 (3 µM) for additional 12 h, as previously done by us (Esposito et al., 2011). Then, the cells have been stimulated with insulin (100 nm) or ISO (10 µM) dissolved in fresh serum-free media. Control unstimulated cells were maintained in serum-free media.

### Immunoblots

Cells (fibroblasts) and left ventricular (LV) samples were lysed in a RIPA buffer with protease (cOmplete-Roche, USA) and phosphatase inhibitor (PhosSTOP-Roche, USA) cocktail (Roche). Protein samples were quantified using a DC^™^ Protein Assay (Bio-Rad) and read at 750 nm using an iMark microplate reader (Bio-Rad). Then proteins (40 µg) were separated by 4–20% Sodium Dodecyl Sulphate - PolyAcrylamide Gel Electrophoresis (SDS-PAGE) (Invitrogen) and were transferred to PVDF membrane (Bio-Rad). After blocking, with milk 5% in TBS-Tween 0.1%, the membranes were incubated and probed with the first antibody at 4°C overnight according to manufacturer’s instructions. Then, the proteins were probed with a corresponding secondary antibody, followed by the visualization of the proteins with a chemi-doc XRS system (Bio-Rad), and quantitative densitometric analysis was performed using the chemi-doc XRS imaging software. Protein levels of a) GRK2 (sc-562, C-15, Santa Cruz Biotechnology, 1:1,000; 05-465, Millipore, 1:1,000); b) GAPDH (sc-32233, 6C5, Santa Cruz Biotechnology, 1:2,000); c) phosphor-ERK (extracellular signal-regulated kinase) 1/2 (#9106, Cell Signaling, 1:1,000), total ERK 1/2 (#9102, Cell Signaling, 1:1,000); d) phospho-Akt (pAkt, sc-514032, Santa Cruz Biotechnology, 1:1,000); e) total Akt (sc-8312, Santa Cruz Biotechnology, 1:1,000); f) phospho-IRS1 (^Ser307^pIRS1, #05-1087, Millipore, 1:1,000); g) total IRS1 (tIRS1, sc-515017, Santa Cruz Biotechnology, 1:1,000); and h) β-1 adrenergic receptor (β1AR, PA1-049, Invitrogen, 1:1,000) were assessed.

### Cyclic Adenosine 3′,5′-Monophosphate (cAMP) Assay

cAMP levels were assessed using a commercial kit (Cayman chemical—501001), following the manufacturer’s instructions. Briefly, 3T3-L1 fibroblasts were plated in six-well multi-well, and after stimulation, the cells were lysed in 250 μl of 0.1-M HCl. Then, after centrifugation (1,000 g for 10 min), the resulting supernatants were quantified with a DC^™^ Protein Assay (Bio-Rad) to assess equal protein concentration. Then, the plate was read at 415 nm using an iMark microplate reader (Bio-Rad). Fifty microliters of the lysate were used to perform the enzyme-linked immunosorbent assay (ELISA) assay.

### Animal Models

All animal procedures were performed in accordance with the guidelines of the Institutional Animal Care and Use Committee of Temple University Lewis Katz School of Medicine. We used wild-type (WT) mice, mice bearing floxed grk2 (grk2-fl/fl), and cardiac-specific GRK2 knockout (KO) mice, generated breeding αMHC-Cre mice with grk2-fl/fl (Cannavo et al., 2016). All animals (males and females, 9–10 weeks) were maintained on a C57Bl/6 background.

**Experimental Procedure:** *Pump implantation*—Mice were anesthetized with isoflurane [2.5% (vol/vol)] and mini-osmotic pumps (1004, ALZET, DURECT Co., Cupertino, USA), loaded with Phosphate-buffered saline (PBS) and 5% ethanol (vehicle); aldosterone (2 μg·mouse·day^−1^) and spironolactone (20 mg·kg^−1^·day^−1^) were implanted subcutaneously through a subscapular incision, which was then closed using 3.0 silk suture (Ethicon), as previously described (Cannavo et al, 2016; Tian et al., 2009). After 4 weeks of infusion, transthoracic echocardiography was performed, and then blood samples were collected by puncturing the heart, and after euthanasia, the heart was explanted and excised for pathological examination and immunohistochemistry.

*Myocardial Infarction (MI)*—Surgically induced MI was performed in WT mice (C57BL6, 9-week-old both females and males) as previously described (Gao et al., 2010). One-day post-MI, mice were randomly assigned to one of the following groups: MI and MI + spironolactone (MI + Spiro). Sham-operated animals were used as control.

### Echocardiography

Transthoracic echocardiography was performed in all experimental groups (vehicle and aldosterone infused; sham-operated, MI, and MI + Spiro) to assess cardiac structure and function at baseline and at the end of the study period (4 weeks), using a VisualSonics VeVo 2100 system (VisualSonics, Toronto, Canada), as previously described (Cannavo et al., 2016). No gender-dependent differences were observed in terms of basal, post-MI, or post-aldosterone cardiac function.

### Histology

Cardiac specimens were fixed in 4% formaldehyde and embedded in paraffin. After deparaffinization and rehydration, 5-μm-thick sections were prepared and mounted on glass slides.

*Picro-Sirius Red Staining*—Cardiac sections were stained with 1% Sirius Red in picric acid (Sigma-Aldrich, St. Louis, Missouri) to detect interstitial fibrosis and to calculate infarct size (Cannavo et al., 2017). The percentage of fibrosis and the infarct size were quantified using a software (ImageJ). Cardiac fibrosis images were acquired using a BA410 microscope (Motic^®^). For each of the samples, five to six fields (∼400–900 cells for field) were acquired to detect fibrotic areas.

*TUNEL staining*—Terminal deoxynucleotidyl transferase-mediated dUTP nick-end labeling (TUNEL) was performed on fixed paraffin-embedded LV sections using a commercial kit (Roche), and the assay was performed according to manufacturer’s instructions. Finally, cardiac sections were mounted with Fluoroshield with 4′,6-diamidino-2-phenylindole (DAPI) (Sigma-Aldrich, St. Louis, Missouri). Fluorescent images were acquired using a ZOE^™^ Fluorescent Cell Imager (Bio-Rad). For each of the samples, five to six fields (∼400–900 cells for field) were acquired.

## Analysis and Statistics

All experimental procedures (*in vitro* and *in vivo*) were performed in a blinded fashion. Data are expressed as means ± SE. Statistical significance was determined by a Student *t*-test or Mann–Whitney *U* exact test when the sample size was <10. For multiple comparisons, one-way analysis of variance with Dunnet’s or Tukey’s *post hoc* tests was performed. All data were analyzed using GraphPad Prism software version 8 (GraphPad Software, La Jolla, California). Statistical significance was accepted at *p* < 0.05.

## Results

### GRK2 Signaling Downstream of Aldosterone/MR System Impairs Insulin Signaling in 3T3 Fibroblasts

**Figure 1:**
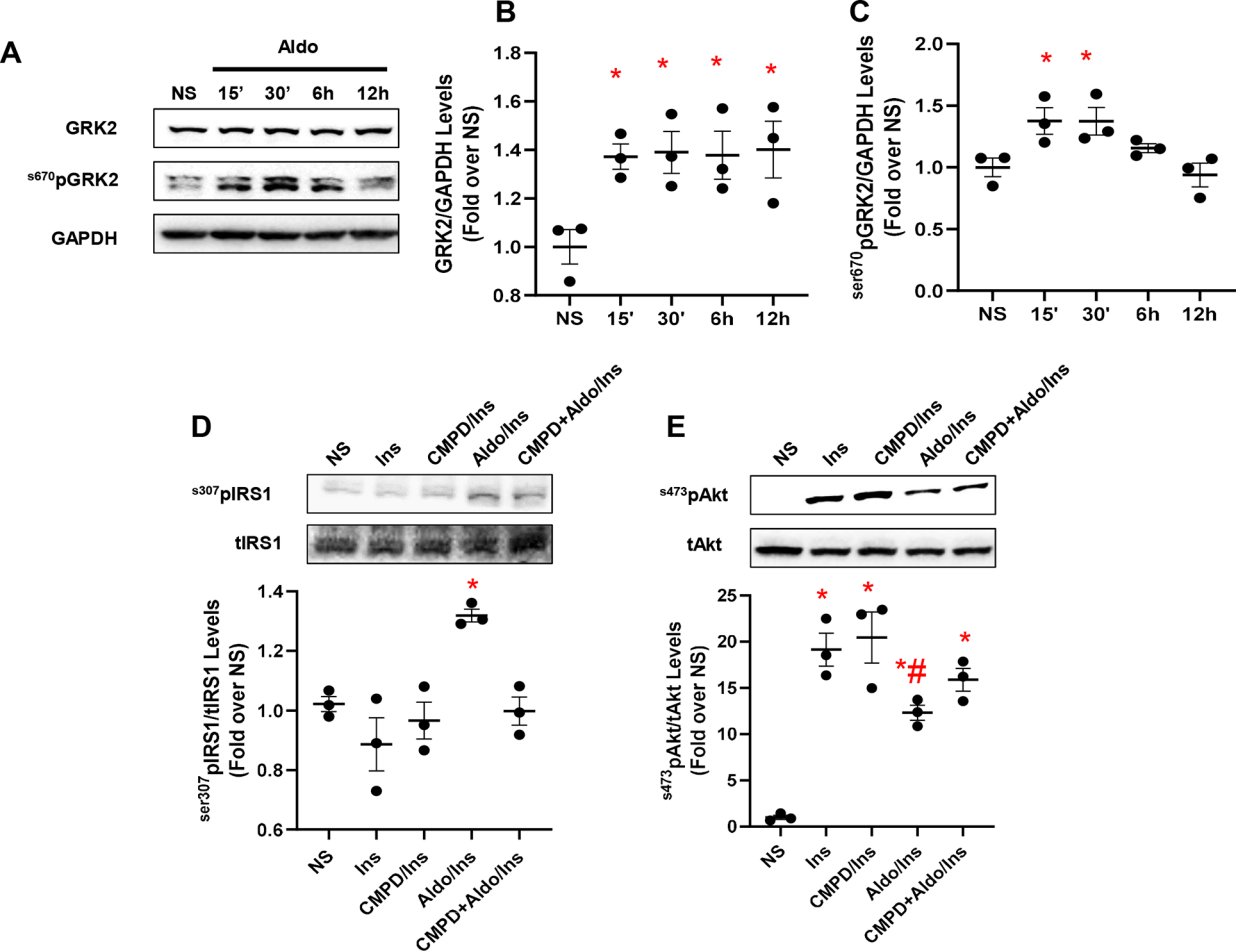
Aldosterone impairs insulin signaling *in vitro* in fibroblasts. Representative immunoblots **(A)** and densitometric quantitative analysis **(B–C)** of multiple independent experiments (*n* = 3) to evaluate GRK2 phosphorylation (Ser670) and expression levels, in 3T3 fibroblasts. Shown is a time course (15 min to 12 h) of aldosterone (Aldo, 1 µM) treatment; GAPDH was used as loading control; Dunnet’s *post hoc* test. *p < 0.05 vs unstimulated cells (NS). **(D)** Representative immunoblots (upper panels) and densitometric quantitative analysis (lower panel) of multiple independent experiments (*n* = 3) to evaluate IRS1 phosphorylation levels (^s307^pIRS1) as a ratio of inactivated IRS1 to total IRS1 (tIRS1). The cells were either NS or stimulated with Aldo (1 µM) and/or CMPD101 (3 µM) for 12 h. After Aldo and/or CMPD101 treatment, cells were stimulated with insulin (Ins, 100 nM) for 15 min. Tukey’s *post hoc* test. *p < 0.05 vs NS. **(E)** Representative immunoblots (upper panels) and densitometric quantitative analysis (lower panel) of multiple independent experiments (*n* = 3) to evaluate Akt phosphorylation (^s473^pAkt) as a ratio of activated Akt to total Akt (tAkt), in cells either NS or stimulated with Aldo (1 µM) and/or CMPD101 (3 µM) for 12 h. Then, cells were stimulated with Ins (100 nM) for 15 min. Tukey’s *post hoc* test. *p < 0.05 vs NS; #*p* < 0.05 vs Ins.

In adipocytes and in VSMCs, aldosterone blocks insulin signaling, contributing to insulin resistance (Hitomi et al., 2007; Wada et al., 2009). Yet, whether GRK2 has a role in this chain of events remains untested. Therefore, to fill this gap in knowledge, we stimulated 3T3 cells with aldosterone (1 µM) at different time points (15 min, 30 min, 6 h, and 12 h) to analyze the expression levels of GRK2. Surprisingly, GRK2 protein levels were increased as early as 15 min after the aldosterone stimulation initiation, and they remained elevated at 30 min, 6 h, and 12 h, compared with those in unstimulated cells (NS) that exhibited no change in the expression of this kinase between these same time points (**Figures 1A, B**). Of note, mRNA transcript levels for GRK2 were not elevated over control levels 15–30 min or 6 h after aldosterone stimulation (**Supplementary Figure 1**). A marked elevation in GRK2 mRNA levels was evident only after 12 h of aldosterone stimulation (**Supplementary Figure 1**). This evidence is entirely consistent with previous reports from Cipolletta and coworkers (Cipolletta et al., 2009) who demonstrated that, following acute ISO or insulin stimulation, GRK2 is markedly upregulated, thus corroborating the notion that this kinase is critical for the regulation of physiological pathways governed by either GPCR- or non-GPCR-related factors. Furthermore, we have previously reported that 30-min stimulation with aldosterone is sufficient to phosphorylate GRK2 at the serine 670 site (^s670^pGRK2), inducing the translocation of the kinase to the mitochondria. On these grounds, we next analyzed the phosphorylation levels of GRK2 and found that these levels varied with time (**Figure 1C**). More in detail, from 15 to 30 min after aldosterone stimulation initiation, ^s670^pGRK2 amounts were markedly elevated over control levels. However, already 6 or 12 h after aldosterone superfusion, the extent of ^s670^pGRK2 sharply declined (**Figure 1C**). In aggregate, this set of data suggests that, upon aldosterone stimulation, GRK2 is able to deploy both its more canonical effects, such as regulating plasma membrane receptor function, i.e., βAR signaling, and noncanonical actions, such as those due to its translocation to mitochondria where the kinase can bind to non-GPCR-related molecular entities (Pitcher et al., 1999; Cannavo et al., 2018b).

Based on the previously reported findings, we next set out to evaluate the impact of aldosterone/GRK2-dependent signaling on insulin and βAR receptor-dependent pathways. To this end, a group of cells was pretreated with the GRK2 inhibitor, CMPD101 (3 µM) (Lowe et al., 2015; Bouley et al., 2017), in the presence or absence of aldosterone (1 µM). Then, these cells were stimulated with insulin (100 nM, for 15 min) and tested for the phosphorylation levels of IRS1, at the serine 307 (^s307^pIRS1). This is a well-consolidated site targeted by GRK2 that, upon phosphorylation, shuts down the insulin signaling (Ciccarelli et al., 2011). We also analyzed the activation of Akt by monitoring its phosphorylation at serine 473 (^ser473^pAkt). As shown in **Figures 1D, E**, as compared with unstimulated cells (NS), insulin did not increase the amount of ^s307^pIRS1 but induced a sustained activation of Akt. In stark contrast, aldosterone treatment markedly elevated ^s307^pIRS1 levels while reducing Akt activity of Akt in response to insulin (**Figures 1D, E**). Of relevance, the detrimental effects exerted by aldosterone on insulin signaling were blunter by pretreating cells with the GRK2 inhibitor, CMPD101 (**Figures 1D, E**). It is worth stressing that neither CMPD101 nor aldosterone (taken singularly) had any influence on the mediators (Akt) of insulin signaling (**Supplementary Figures 2A, B**).

Finally, we tested the impact of spironolactone, an MR antagonist, previously found to block the upregulation of GRK2 as well as its deleterious effects in response to the activation of aldosterone/MR system (Cannavo et al., 2016). To this aim, a group of 3T3 cells was pretreated with spironolactone (10 µM for 30 min) prior to aldosterone and/or insulin treatment. Insulin stimulation induced a robust Akt activation, and the treatment with the mineralocorticoid hormone blunted this effect (**Supplementary Figure 3A**).

### Aldosterone Alters βAR Signaling in 3T3 Fibroblasts *via* GRK2 Activation

**Figure 2:**
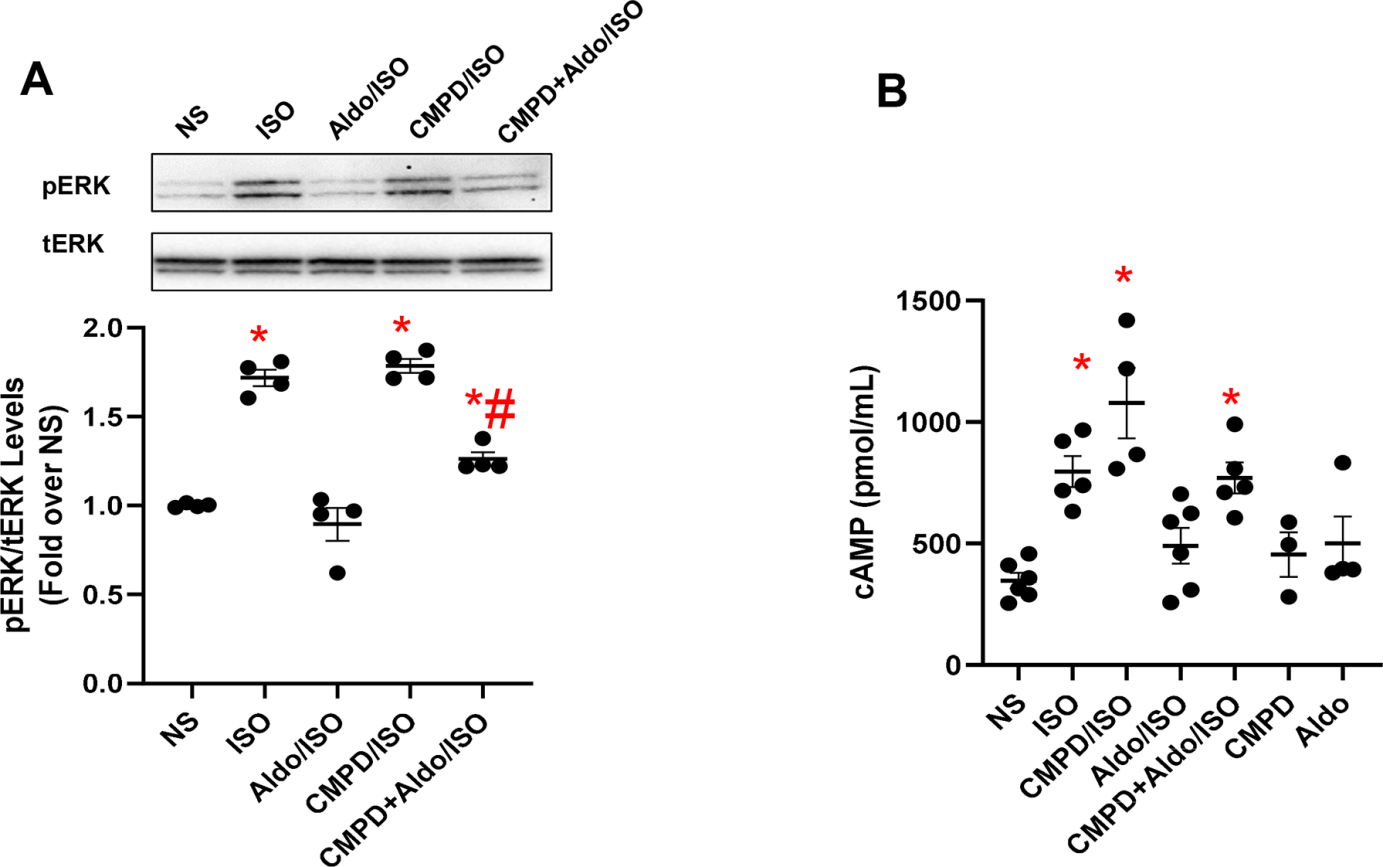
Aldosterone dysregulates βAR signaling *in vitro* in fibroblasts. **(A)** Representative immunoblots (upper panels) and densitometric quantitative analysis (lower panel) of multiple independent experiments (*n* = 4) to evaluate extracellular signal-regulated kinase (ERK) 1/2 phosphorylation (pERK) as a ratio of activated ERK to total ERK (tERK). The cells were either NS or stimulated with aldosterone (Aldo, 1 µM) and/or CMPD101 (3 µM) for 12 h. After Aldo and/or CMPD101 treatment, cells were stimulated with isoproterenol (ISO, 10 µM) for 15 min. Tukey’s *post hoc* test. *p < 0.05 vs NS; #*p* < 0.05 vs ISO. **(B)** Dot plots showing levels of cyclic adenosine 3′,5′-monophosphate (cAMP, pmol/ml) in 3T3 fibroblasts NS or stimulated with ISO (10 µM) for 15 min. Prior ISO stimulation some groups of cells were pretreated with Aldo (1 µM) and/or CMPD101 (3 µM) for 12 h. Tukey’s *post hoc* test. *p < 0.05 vs NS.

A correlation exists between aldosterone and βAR activation (Reddy et al., 2015; Hori et al., 2017). More in detail, in experimental models of LV dysfunction based on the chronic infusion of ISO, either spironolactone or eplerenone effectively prevents ISO-triggered collagen deposition (Reddy et al., 2015; Hori et al., 2017). Interestingly, none of these studies, however, have tested whether MR antagonism also prevents βAR dysfunction due to chronic ISO administration as well as whether chronic MR activation has an impact on βAR function and GRK2 upregulation. To fill these critical gaps in knowledge, we examined the effect of aldosterone on βAR function *in vitro*, using 3T3 cells, assessing the activation status of the ERK that is a useful marker of βAR signaling (Cannavo et al., 2018b). To this end, the cells, either unstimulated (NS) or stimulated with ISO (10 µM for 10 min), were pretreated with aldosterone (1 µM for 12 h) and/or with CMPD101 (3 µM). As shown in **Figure 2A**, in response to ISO, we observed a robust ERK activation (pERK) compared with NS cells, which was blocked entirely by aldosterone pretreatment. Nonetheless, pretreatment of cells with CMPD101 significantly abolished the effects of aldosterone and resulted in enhancement of ERK activation in response to ISO. Next, we analyzed the effects of aldosterone on the production of cAMP that is typically elevated after βAR stimulation (Cannavo et al., 2018b). Importantly, we observed that ISO induced a significant rise in cAMP levels, but this effect was abolished by aldosterone pretreatment (**Figure 2B**). Further, the inhibition of GRK2 had a positive influence on the cAMP formation, thus preventing the harmful effects of aldosterone on βARs. Interestingly, neither CMPD101 nor aldosterone alone had a sizable impact on ERK phosphorylation (**Supplementary Figures 4A, B**) and cAMP formation/accumulation (**Figure 2B**). These data support the notion that aldosterone impairs βAR function and that GRK2 is a crucial component of aldosterone-mediated adverse effects.

### Chronic *in Vivo* Infusion of Aldosterone Upregulates GRK2 in the Heart

**Figure 3:**
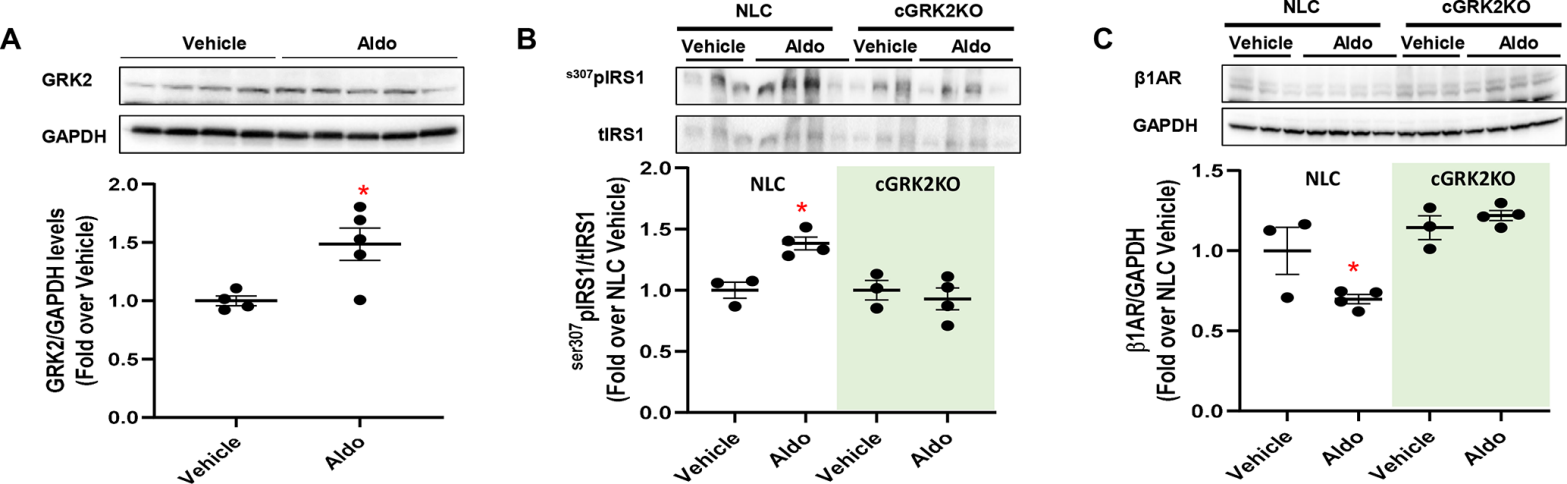
*In vivo* effects of chronic aldosterone infusion on murine myocardium. **(A)** Representative immunoblots (upper panels) and densitometric quantitative analysis (lower panel) showing levels of GRK2 in total cardiac lysates from mice treated with Aldosterone (Aldo, 2 µg·mouse·day^-1^; *n* = 5) or Vehicle (PBS and EtOH 5%; *n* = 4) for 4 weeks. GAPDH levels were used as loading control; Mann–Whitney test. *p < 0.05 vs Vehicle. **(B–C)** Representative immunoblots (upper panels) and quantitative data showing levels of **(B)** phosphorylated IRS1, at serine 307 (^s307^pIRS1) and **(C)** β1-adrenergic receptor (β1AR) in total cardiac lysates from NLC and cardiac GRK2KO mice either treated with Aldo (2 µg·mouse·day^−1^; *n* = 4) or Vehicle (PBS and EtOH 5%; *n* = 3) for 4 weeks. GAPDH levels were used as loading control; Dunnet’s *post hoc* test. *p < 0.05 vs NLC Vehicle.

Hyperaldosteronism is a condition that usually precedes or occurs after MI, precipitating LV dysfunction and remodeling (Weber 2001; He et al., 2011; Cannavo et al., 2016). Importantly, chronic (4 weeks) administration of aldosterone (2 µg·mouse·day^-1^) results in a significant increase in myocyte GRK2 expression and mitochondrial activity, along with augmented cell death and fibrosis, as recently shown by our group (Cannavo et al., 2016). Yet, whether Aldo impairs insulin signaling and βAR function in the mouse heart, *in vivo*, remains to be directly tested. Thus, to translate our *in vitro* findings to *in vivo* clinically relevant situations, we subjected Normal Littermate Control (NLC) (WT) and cardiac-specific GKR2 knockout (cGRK2KO) mice to a 4-week continuous infusion of aldosterone. A group of mice was infused with vehicle solution as a control. We found that in NLC mice, this intervention elicited a marked rise in GRK2 protein levels in LV lysates, as compared with those in vehicle-treated animals (**Figure 3A**). This change was accompanied by a prominent surge in cardiac ^ser307^pIRS1 levels and by marked downregulation of β1AR density (**Figures 3B, C**). Importantly, the deleterious effects associated with aldosterone stimulation were utterly blunted in cGRK2KO mice (**Figure 3**). Indeed, in the presence of low GRK2 expression (**Supplementary Figure 5**), aldosterone stimulation failed to either increase ^ser307^pIRS1 levels or reduce β1AR density. Thus, these data attest that GRK2 is chiefly involved in the *in vivo* signaling effects elicited by chronic aldosterone stimulation.

### MR Antagonism by Spironolactone Prevents Postischemic Cardiac Insulin Resistance and β1AR Dysfunction Thus Preventing HF Onset

MR blockade, either *via* spironolactone or eplerenone treatment, prevents Aldo-induced increment in GRK2 expression and activity (Cannavo et al., 2016). However, whether spironolactone exerts its protective, anti-ischemia effects, at least in part, through the inhibition of GRK2, and thus restoring cardiac insulin and/or β1AR sensitivity, is currently unknown.

**Figure 4:**
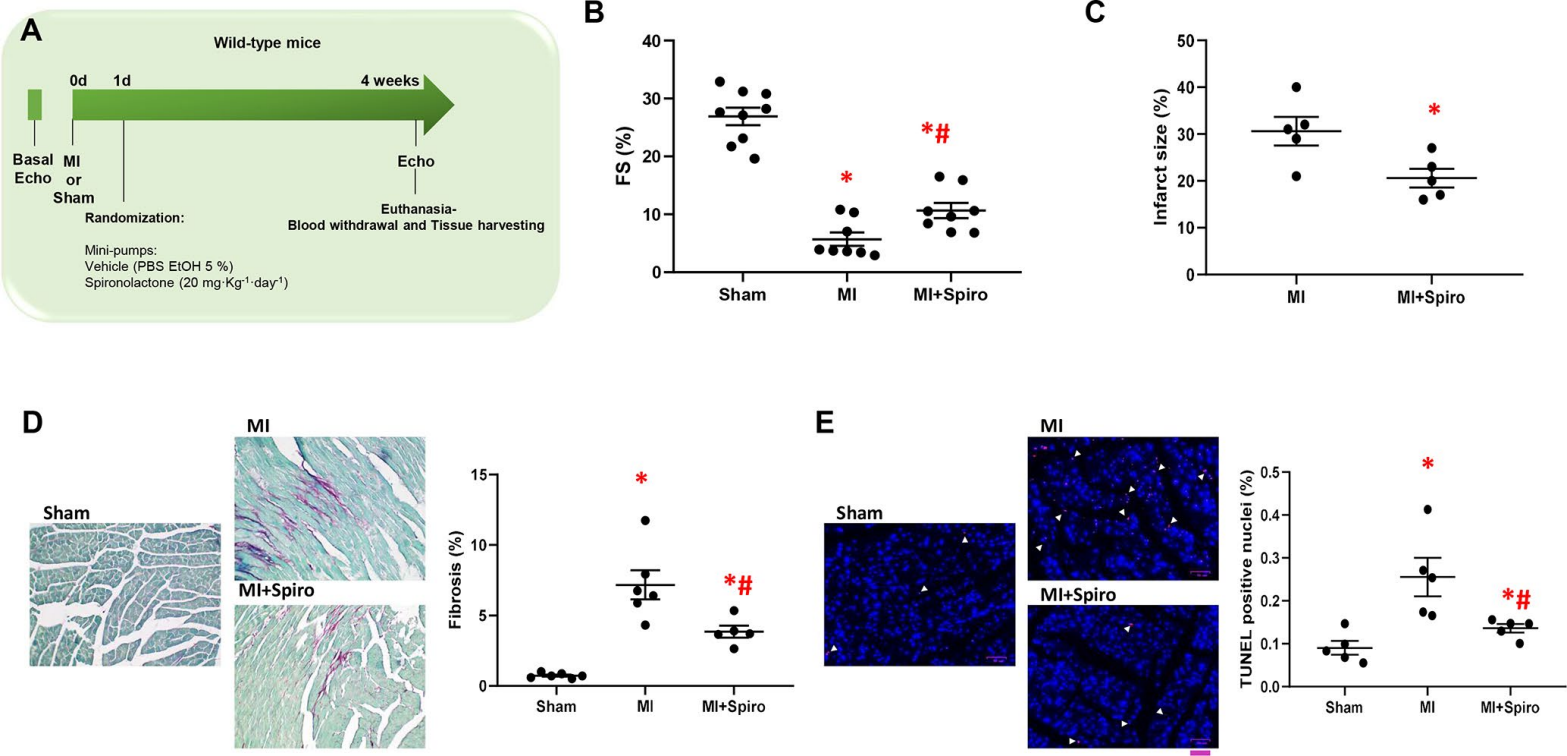
Spironolactone attenuates cardiac dysfunction and prevents adverse remodeling in post-ischemic murine hearts. **(A)** Schematic representation of 4-week study of myocardial infarction (MI) in mice. Echocardiography was performed at day 0 (0d), and MI were surgically induced. Sham-operated mice were used as controls. After 1-day (1d) post-MI, mice were randomized for treatments: MI (controls), MI + Spironolactone (Spiro, 20 mg·kg^−1^·day^−1^). Four week post-MI, echocardiography was performed and animals (*n* = 8–9 per group) euthanized for tissue harvesting. **(B)** Dot plots showing measurements for fractional shortening (FS, %). Tukey’s *post hoc* test. *p < 0.05 vs Sham; #*p* < 0.05 vs MI. **(C)** Dot plots showing percentage of infarct size in MI, MI + Spiro mice (*n* = 5 mice per group). Mann–Whitney test. *p < 0.05 vs MI. **(D**–**E)** Representative images and quantitative data showing percentage of **(D)** cardiac fibrosis (Picro-Sirius red staining, magnification 20×) and **(E)** cell death (TUNEL (terminal deoxynucleotidyl transferase-mediated dUTP nick-end labeling)/DAPI staining, scale bar: 50 µM) in cardiac sections from Sham, MI, and MI + Spiro mice (*n* = 4–6 mice per group). Tukey’s *post hoc* test. *p < 0.05 vs Sham; #*p* < 0.05 vs MI.

To address these questions, MI was surgically induced in mice *via* coronary artery ligation (Gao et al., 2010). One-day after MI, mice were treated with spironolactone (20 mg·kg·day^−1^) for the entire study period (4 weeks). Sham-operated animals were used as controls. The echocardiographic analysis was performed at baseline and 4 weeks post-MI (**Figure 4A**).

**Figure 5:**
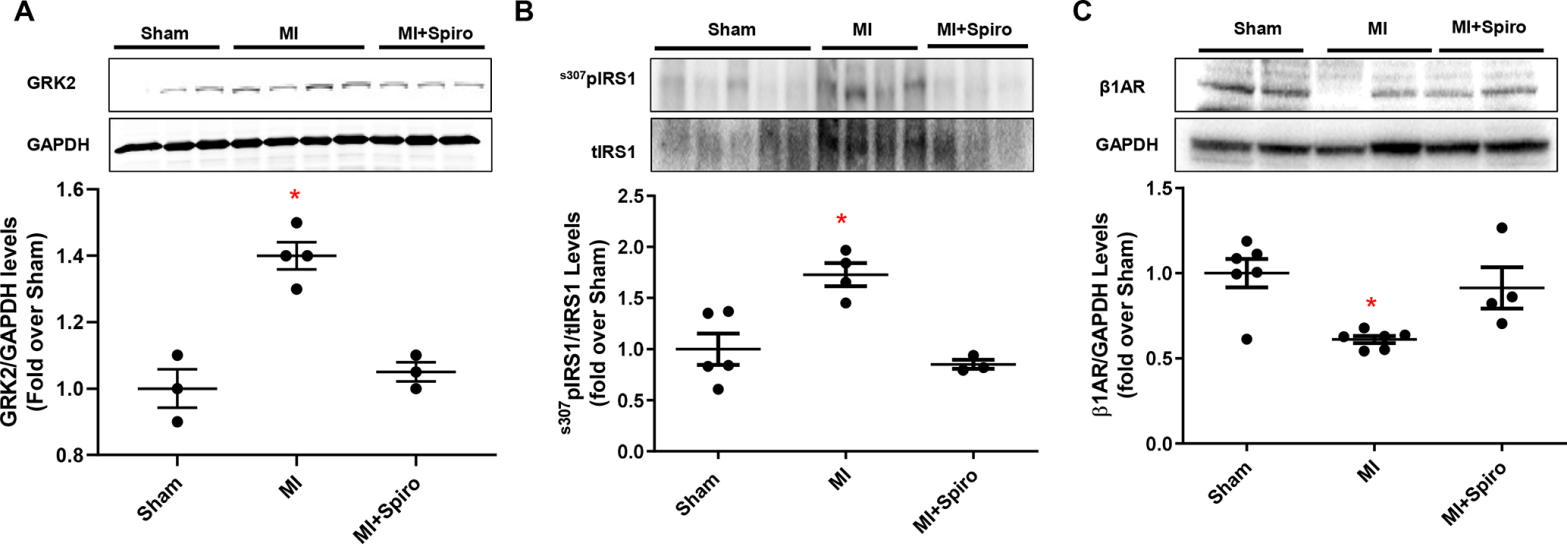
Aldosterone/mineralocorticoid receptor (MR) blockade prevents GRK2 upregulation in postischemic failing hearts and abolishes Insulin and βAR signaling downregulation. **(A–C)** Representative immunoblots (upper panels) and quantitative data (lower panels) showing levels of GRK2 **(A)**, ^ser307^pIRS1 **(B)** and β1AR **(C)** in total cardiac lysates of Sham, MI, and MI + Spiro mice (*n* = 3–6 mice per group). tIRS1 and GAPDH levels were used as loading control; Tukey’s *post hoc* test. *p < 0.05 versus Sham.

As shown in **Figure 4B**, MI-inflicted mice displayed a marked deterioration of LV function compared with Sham animals, as indexed by percentage fractional shortening (%FS). Conversely, spironolactone-treated MI mice had significantly ameliorated LV dysfunction and reduced infarct size (**Figures 4B, C**). In a consistent manner, at the tissue level, myocytes from untreated MI mice harbored prominent cardiac fibrosis, assessed by picro-sirius red staining (**Figure 4D**), and augmented myocardial cell death, as per TUNEL staining (**Figure 4E**). It is worth noting that all these adverse effects were significantly blunted in MI mice receiving spironolactone (**Figures 4D, E**). Intriguingly, at the molecular level, the marked MI-dependent GRK2 upregulation was prevented by this MR antagonist (**Figure 5A**). Consistently, augmented levels of ^ser307^pIRS1 and reduced β1AR expression, accompanying the increased GRK2 activity and attesting insulin signaling dysfunction and β1AR downregulation, were also significantly attenuated by spironolactone (**Figures 5B, C**). Thus, MR antagonism by spironolactone ameliorates post-MI LV dysfunction, improving, among other possible beneficial effects, insulin resistance and β1AR dysfunction.

## Discussion

SNS and RAS are well-recognized primary pathogenic drivers of HF (Swedberg et al., 1990). Accordingly, most of the agents that block the activation of these systems are part of the current HF therapeutic armamentarium [i.e. β-blockers, Angiotensin II receptor (AT1R) blockers, ACE inhibitors, and MR antagonists] (Cannavo et al., 2013; D’Addio et al., 2017; Komici et al., 2017). However, since not all patients can benefit from these drugs, there is a keen interest in the identification of additional, specific strategies able to block the hyperactivity of these noxious systems (Shafiq and Miller, 2009). Importantly, MR antagonists have shown to be therapeutic and safe in a wide range of HF patients with reduced EF, both symptomatic (in NYHA class III and IV) (Pitt et al., 1999) and in asymptomatic or mildly symptomatic (in NYHA class I and II) (Zannad et al., 2011). Indeed, these therapeutic agents are currently indicated in the majority of HF patients; thus, the interest in the mechanism beyond MR inhibition is continuously growing.

**Figure 6:**
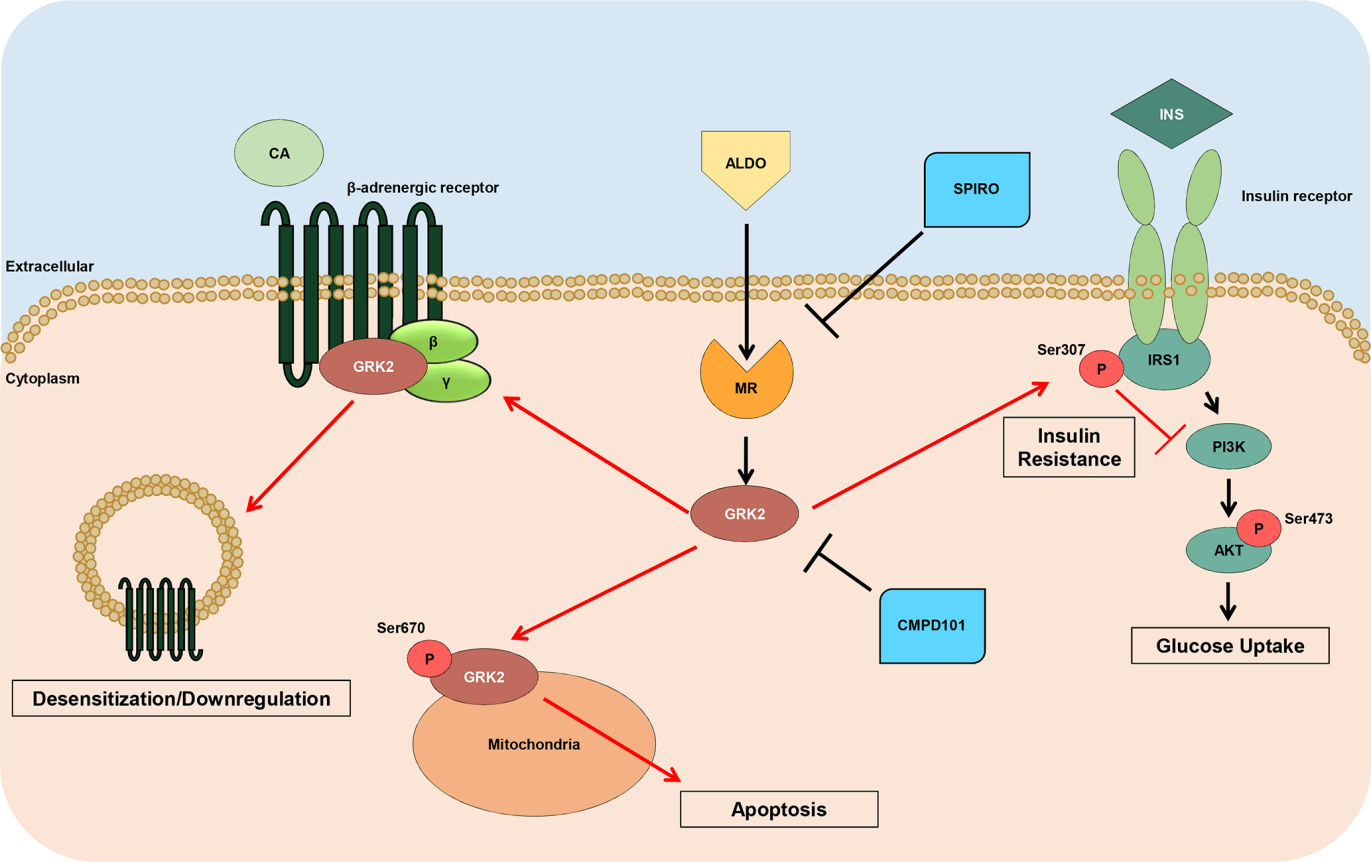
Schematic representation of GRK2 noxious effects downstream Aldosterone. High levels of Aldosterone induce the hyperactivation of MR leading to the upregulation of GRK2, which in turn, induces the negative phosphorylation of IRS1 (^ser307^pIRS1), with a consequent impaired response to insulin (INS) and reduced Akt activation, and downregulates βARs that do not response to catecholamine (CA) stimulation. Further, this kinase, when phosphorylated at ser670, is able to translocate to mitochondria, where it increases myocyte apoptosis. Of note, either spironolactone or CMPD101 (GRK2-inhibitor) are able to block the expression and noxious effects of GRK2 downstream aldosterone.

In this context, we have recently revealed novel mechanisms whereby aldosterone may affect cardiac function (Cannavo et al., 2016). More in details, we have found that the adverse effects of aldosterone on cardiomyocytes are not exclusively mediated by MR activation but also mediated by GRK2 (Cannavo et al., 2016). Importantly, this kinase is centrally involved in MR-mediated cardiac toxicity that is negatively affecting mitochondrial function and survival of cardiomyocytes. Moreover, in the present study, our data add new important pieces to the mosaic of how aldosterone/GRK2 system impacts on cardiac function and remodeling (**Figure 6**). In this context, we have found that aldosterone negatively affects insulin and β1AR function both *in vitro* in 3T3 cells and *in vivo* in the hearts of two murine models of hyperaldosteronism (aldosterone-infused or infarcted mice). Moreover, we have demonstrated that such mechanism appears to be orchestrated by GRK2. More in detail, we have shown in 3T3 cells that soon after (15 and 30 min) aldosterone stimulation initiation, GRK2 becomes upregulated and phosphorylated at s670, which is in line with our previous report in cardiomyocytes (Cannavo et al., 2016). However, GRK2 expression levels remain high even only up to 12 h. After that, the amount of the kinase phosphorylation comes back to control levels. This evidence suggests that, upon phosphorylation, GRK2 is able to both translocate to mitochondria, following phosphorylation, and to bind plasma membrane receptor when unphosphorylated, as previously reported (Pitcher et al., 1999; Cannavo et al., 2016; Cannavo et al., 2018b). In the same vein, here we demonstrate that, after chronic aldosterone treatment of 3T3 cells, a prominent inhibition of insulin signaling occurs, as documented by the increased ^s307^pIRS1 levels and a subsequent reduction in ^s473^pAkt levels, followed by the marked impairment of the βAR function with a consequent decrease of ERK activation and cAMP production in response to ISO stimulation. Interestingly, the usage of the GRK2 inhibitor, CMPD101, markedly blunted the effects of aldosterone, preventing both insulin and βAR signaling dysfunction.

Next, we aimed at confirming the *in vitro* findings in an *in vivo* model of hyperaldosteronism, such as the chronic (4 weeks) continuous infusion of aldosterone in mice, as done previously (Cannavo et al., 2016). With this tool in hand, we demonstrated that aldosterone increased cardiac ^s307^pIRS1 levels and caused a massive β1AR downregulation in NLC mice but not in cGRK2KO mice. This evidence goes hand in hand with previous findings showing that cGRK2KO hearts were functionally and structurally protected against hyperaldosteronism (Cannavo et al., 2016).

Finally, our results that were obtained in infarcted mice with the MR antagonist, spironolactone, which has previously been attested as a blocker of aldosterone-dependent GRK2 upregulation, consolidate the idea of the potential GPCR dependence into the pathological effects induced by aldosterone.

More in details, our data demonstrate that following MI, GRK2 levels are significantly increased while insulin and β1AR signaling pathway are impaired. Conversely, spironolactone treatment is able to mitigate such noxious aldosterone activities and to prevent both IRS1 negative phosphorylation, β1AR downregulation, and GRK2 upregulation. Interestingly, all the beneficial effects of spironolactone correlated with the preserved cardiac function and better remodeling parameters markedly deteriorated by MI.

### Limitations

One limitation of our study is the lack of a randomization parameter for the MI studies. One day after MI, we randomly assigned the mice to spironolactone or vehicle treatment, performing an echocardiographic analysis only at baseline (before MI) and at the end of the study period. Although we did not check the degree of the infarct size in the two groups, at 1-day post-MI, we are confident that the infarct area was homogeneous between the animals. Indeed, we have long-standing expertise on *in vivo* MI, and our method has been proven to inflict a very reproducible infarct size (Gao et al., 2010). Moreover, in studies in which we checked for cardiac function after randomization, we did find a statistically significant difference among post-MI randomized groups (Cannavo et al., 2017). Therefore, the effects seen with the different treatments are not due to an unequal initial magnitude of the infarct lesion. Another limitation of the present study is the lack of data regarding the effects of aldosterone on the β2AR signaling pathway. However, β1AR signaling is the major isoform expressed in cardiomyocytes, liable for the main actions of the βAR system on the cardiac function and tightly regulated by GRK2. Future, in-depth studies shall address the impact of aldosterone on the β2AR signaling in cardiomyocytes, fibroblasts, and endothelial cells. Indeed, this receptor subtype is much less expressed at cardiomyocyte level, but it is also targeted by GRK2; therefore, aldosterone may also alter β2AR-dependent cardiac cell functions.

## Conclusion

Previous evidence has attested that an association exists between aldosterone and insulin signaling dysfunction. However, no studies have assessed the relevance of this cross talk on heart function. Nor have studies clarified whether GRK2, a primary cardiac regulator of IRS1, has any role in the way aldosterone influences the insulin signaling and also in βAR-mediated modulation of myocardial function and response to ischemic stress. Here, we fill these gaps in knowledge by demonstrating, for the first time, that canonical and noncanonical actions of GRK2 account for aldosterone-triggered attenuation of insulin- and βAR-mediated effects at the heart levels. Several clinical cardiovascular disorders are characterized by a persistently elevated “aldosterone tone”; see, for instance, the hypertensive or the HF syndrome. In light of this, the current evidence showing that, in infarcted mice, the MR blocker, spironolactone, offsets aldosterone-induced cardiac insulin-signaling dysfunction and β1AR downregulation by blunting canonical (and likely noncanonical) effects mediated by GRK2 levels suggests the unique opportunity of combining the benefits of a direct MR antagonism to those elicited by the pharmacological inhibition of GRK2, a novel, highly translational perspective that warrants further, in-depth investigation.

## Ethics Statement

All animal procedures were performed in accordance with the guidelines of the Institutional Animal Care and Use Committee of Temple University Lewis Katz School of Medicine.

## Author Contributions

ACa, FM, and GR designed, analyzed data, and wrote the manuscript. ACa, LB, FM, AE, DL, GG, and CP performed the experiments. AR, ACi, NF, NP, and WK revised the manuscript.

## Funding

This research was partly funded by: STAR program 2017 L1— Compagnia San Paolo and Federico II University of Naples and Italian Ministry of Education, Universities and Research- ”Rita Levi Montalcini 2016” (to ACa); CardioPaTh PhD program (to FM); NIH_NHLDBI R01HL136918 and Magic- That-Matters (JHU) (to NP).

## Conflict of Interest Statement

NF and GR provide scientific advice to Istituti Clinici Scientifici ICS Maugeri SpA, Società Benefit, Scientific Institute of Telese Terme, Benevento, Italy.

The remaining authors declare that the research was conducted in the absence of any commercial or financial relationships that could be construed as a potential conflict of interest.
