# Supplementary material for: Aldosterone Jeopardizes Myocardial Insulin and β-Adrenergic Receptor Signaling via G Protein-Coupled Receptor Kinase 2
Source: Front Pharmacol. 2019 Aug 9;10:888. doi: 10.3389/fphar.2019.00888 (PMC6695474; doi:10.3389/fphar.2019.00888)
Supplement: Supplementary file 1 [file Table_1.docx]

**ONLINE SUPPLEMENTARY METHODS**

**Real-Time PCR**

Total RNA was isolated from 3T3 cells with TRIzol (Thermo Fisher Scientific) according to the company’s instructions. After RNA isolation, cDNA was synthesized by reverse transcription of the RNA (iScript cDNA synthesis kit, Bio-Rad Laboratories). Real-time PCR was performed in duplicate on a IQ5 real-time PCR detection system (Bio-Rad Laboratories) using the SYBR Green mix (Bio-Rad Laboratories) and specific primers for mouse GRK2 as follows: forward 5’-CCTGCTCACATCCCTTTTCAAA-3’; reverse 5’-TGTCACTCTCTATGAACTTCTGG -3’ . The expression levels of GRK2 were normalized to the rRNA 18S. Specificity of PCR products was confirmed by melting curve and gel electrophoresis.
