# Supplementary material for: Aldosterone Jeopardizes Myocardial Insulin and β-Adrenergic Receptor Signaling via G Protein-Coupled Receptor Kinase 2
Source: Front Pharmacol. 2019 Aug 9;10:888. doi: 10.3389/fphar.2019.00888 (PMC6695474; doi:10.3389/fphar.2019.00888)
Supplement: Supplementary file 2 [file Table_2.docx]

**SUPPLEMENTARY FIGURE LEGENDS**

**Supplementary Figure 1**

**A)** Bar graph showing quantitative data of Real-time PCR experiments (n=3) to evaluate GRK2 and GAPDH mRNA levels in 3T3 fibroblasts. Cells were either unstimulated (NS) or stimulated with aldosterone (Aldo, 1μM) at different time points (from 15 min to 12 hrs). *p <0.05 vs NS.

**Supplementary Figure 2**

**A)** Representative immunoblots (upper panels) and densitometric quantitative analysis (lower panel) of multiple independent experiments (n=3) to evaluate pAkt/tAkt ratio in cells either unstimulated (NS) or stimulated with aldosterone (Aldo, 1 µM) for 12 h. A group of cells were stimulated with Insulin (Ins, 100 nM) for 15 min. *p <0.05 vs NS. **(B)** Representative immunoblots (upper panels) and densitometric quantitative analysis (lower panel) of multiple independent experiments (n=3) to evaluate pAkt/tAkt ratio in cells either NS or pre-treated with CMPD101 (3 µM) for 12 hrs. A group of cells were stimulated with Ins (100 nM) for 15 min.. *p <0.05 vs NS.

**Supplementary Figure 3**

**A)** Representative immunoblots (upper panels) and densitometric quantitative analysis (lower panel) of multiple independent experiments (n=3) to evaluate pAkt/tAkt ratio in cells either unstimulated (NS) or stimulated with aldosterone (Aldo, 1 µM) for 12 hrs. Prior Aldo stimulation a group of cells was pre-treated with Spironolactone (Spiro, 10 µM) for 30 minutes. After Aldo and/or Spiro treatment, cells were stimulated with Insulin (Ins, 100 nM) for 15 min. *p<0.05 vs NS. #p<0.05 vs Ins.

**Supplementary Figure 4**

**(A)** Representative immunoblots (upper panels) and densitometric quantitative analysis (lower panel) of multiple independent experiments (n=3) to evaluate pERK/tERK ratio in cells either unstimulated (NS) or stimulated with aldosterone (Aldo, 1 µM) for 12 hrs. A group of cells were stimulated with isoproterenol (ISO, 10 µM) for 15 min. *p <0.05 vs Ns**. (B)** Representative immunoblots (upper panels) and densitometric quantitative analysis (lower panel) of multiple independent experiments (n=3) to evaluate pERK/tERK ratio in cells either NS or treated with CMPD101 (3 µM) for 12 h. A group of cells were stimulated with ISO (10 µM) for 15 min. *p <0.05 vs NS.

**Supplementary Figure 5**

**(A)** Representative immunoblots (upper panels) and densitometric quantitative analysis (lower panel) showing GRK2 basal levels in total cardiac lysates from NLC and cardiac-specific GRK2 knockout (cGRK2KO) mice (n=3 per group). GAPDH levels were used as loading control; *p <0.05 vs NLC.
