# Supplementary figures and images for: Aldosterone Jeopardizes Myocardial Insulin and β-Adrenergic Receptor Signaling via G Protein-Coupled Receptor Kinase 2
Source: Front Pharmacol. 2019 Aug 9;10:888. doi: 10.3389/fphar.2019.00888 (PMC6695474; doi:10.3389/fphar.2019.00888)

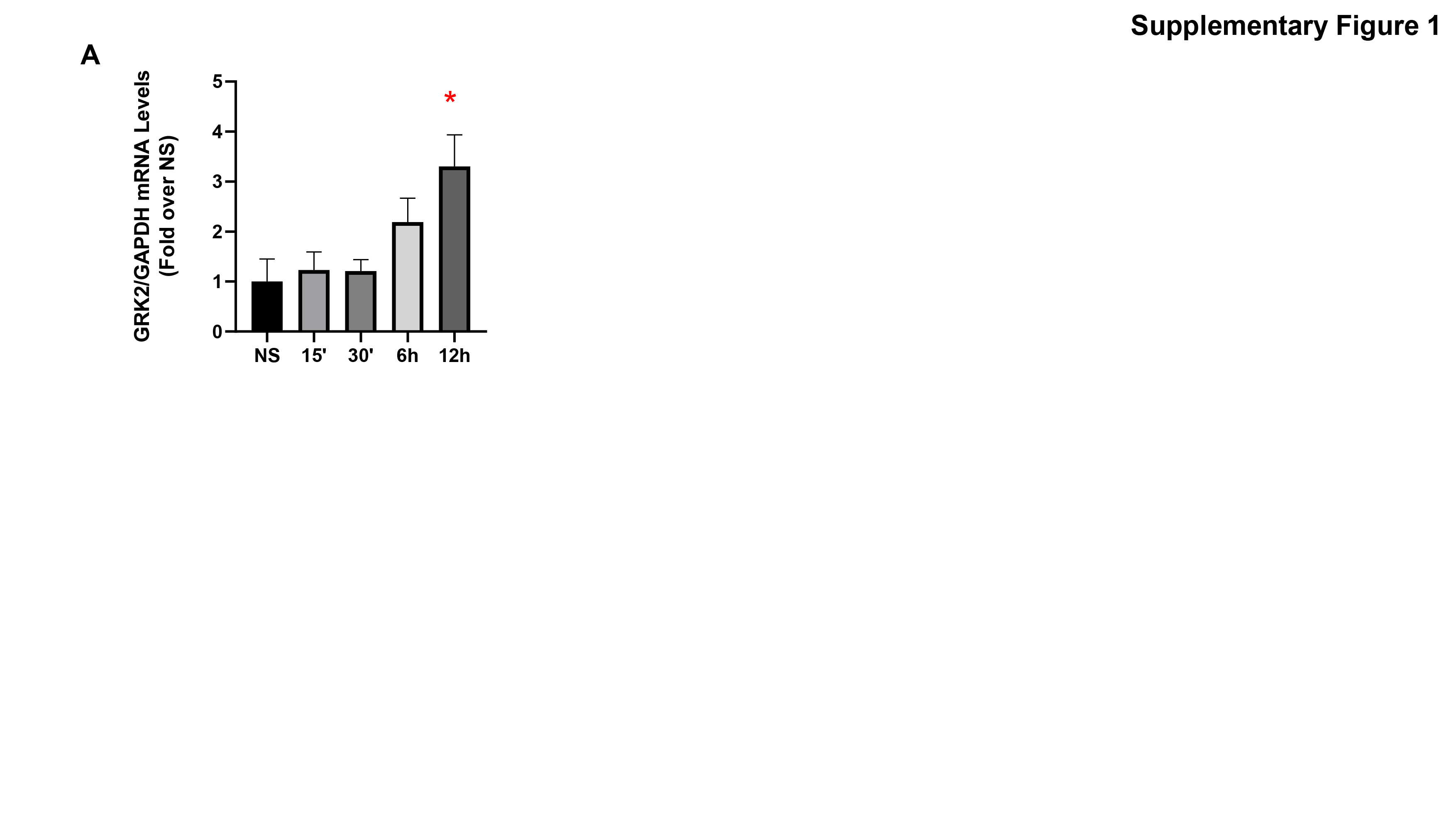

Supplement: Supplementary file 3 [file Image_1.tif]

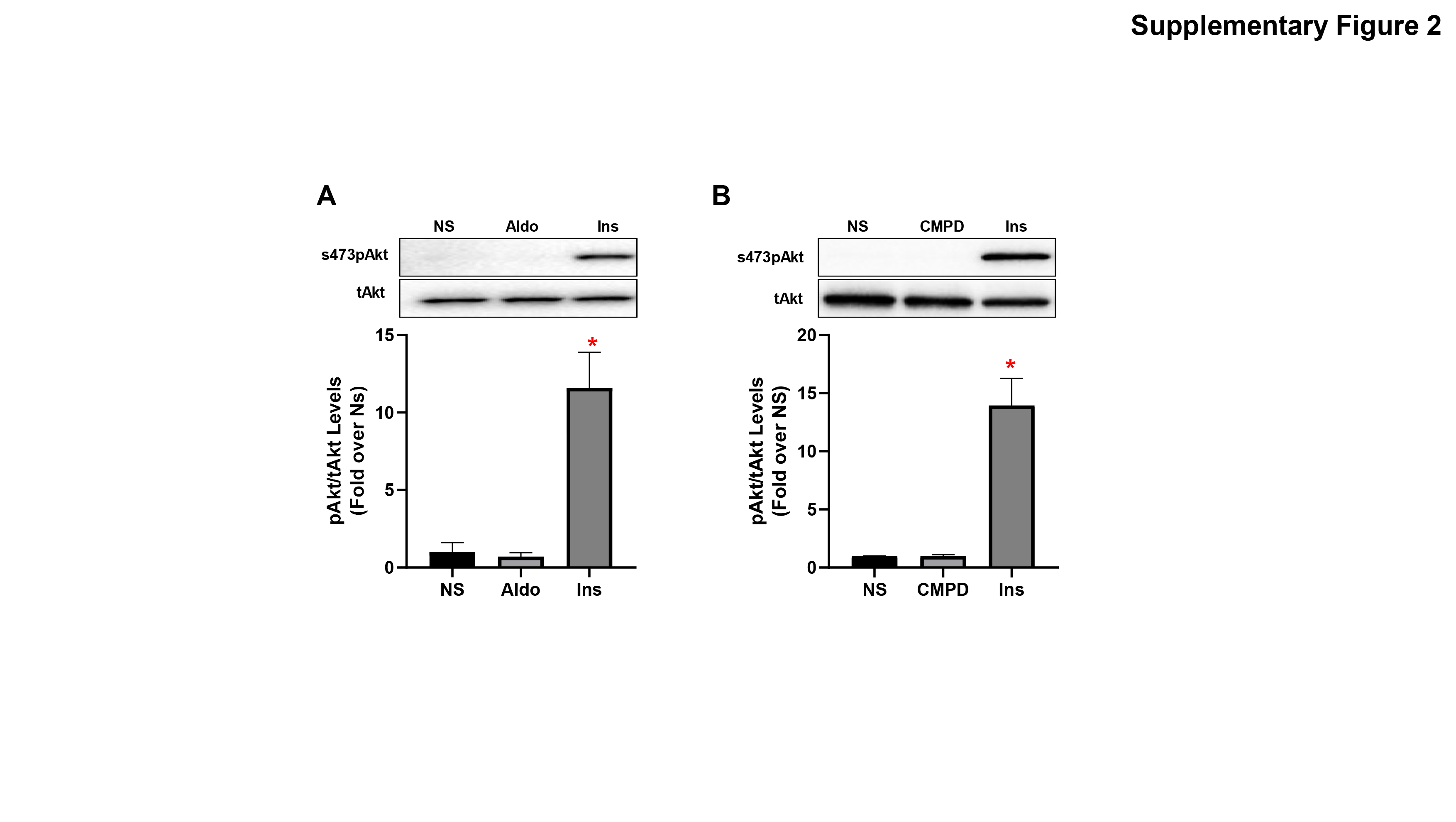

Supplement: Supplementary file 4 [file Image_2.tif]

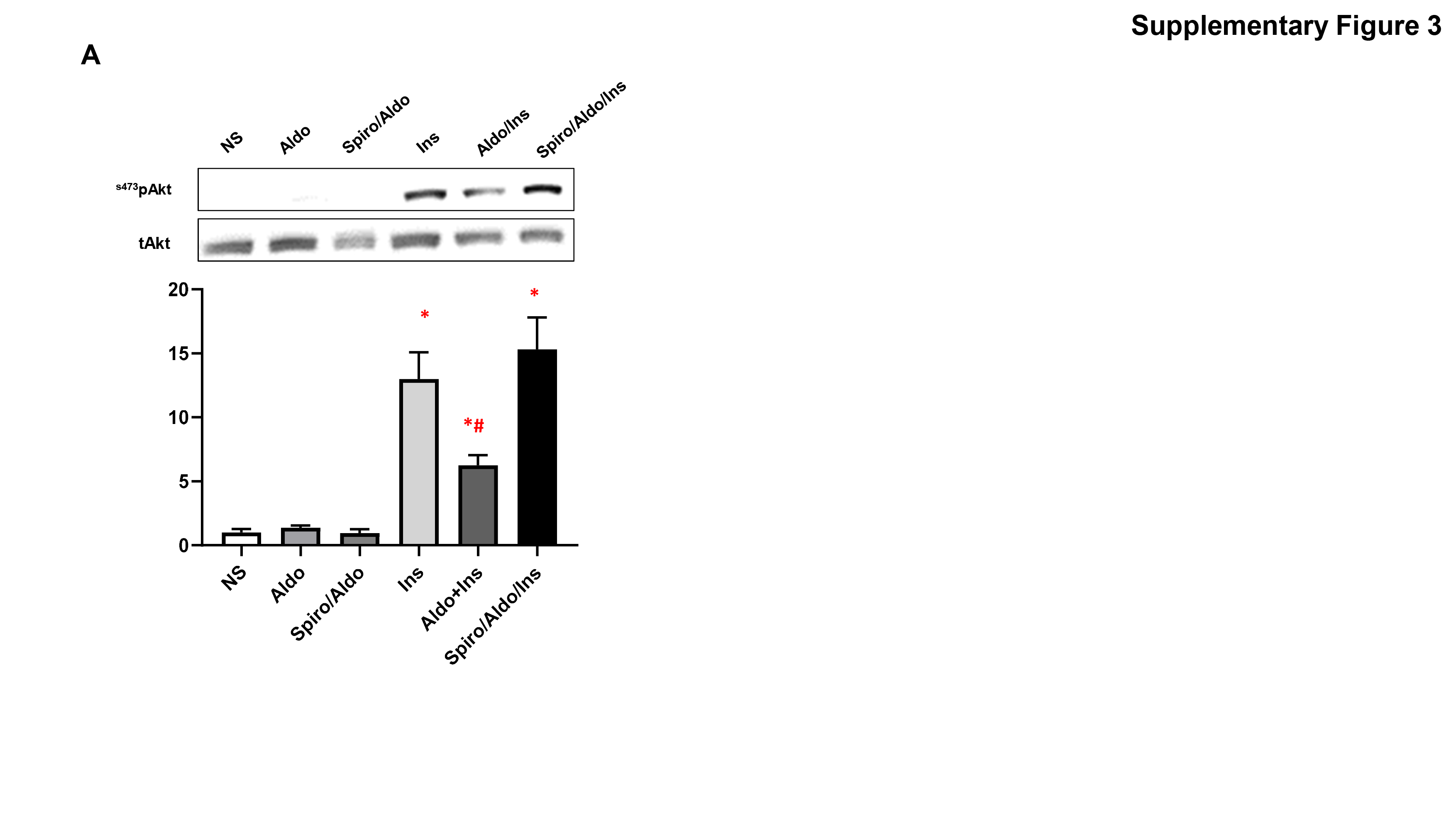

Supplement: Supplementary file 5 [file Image_3.tif]

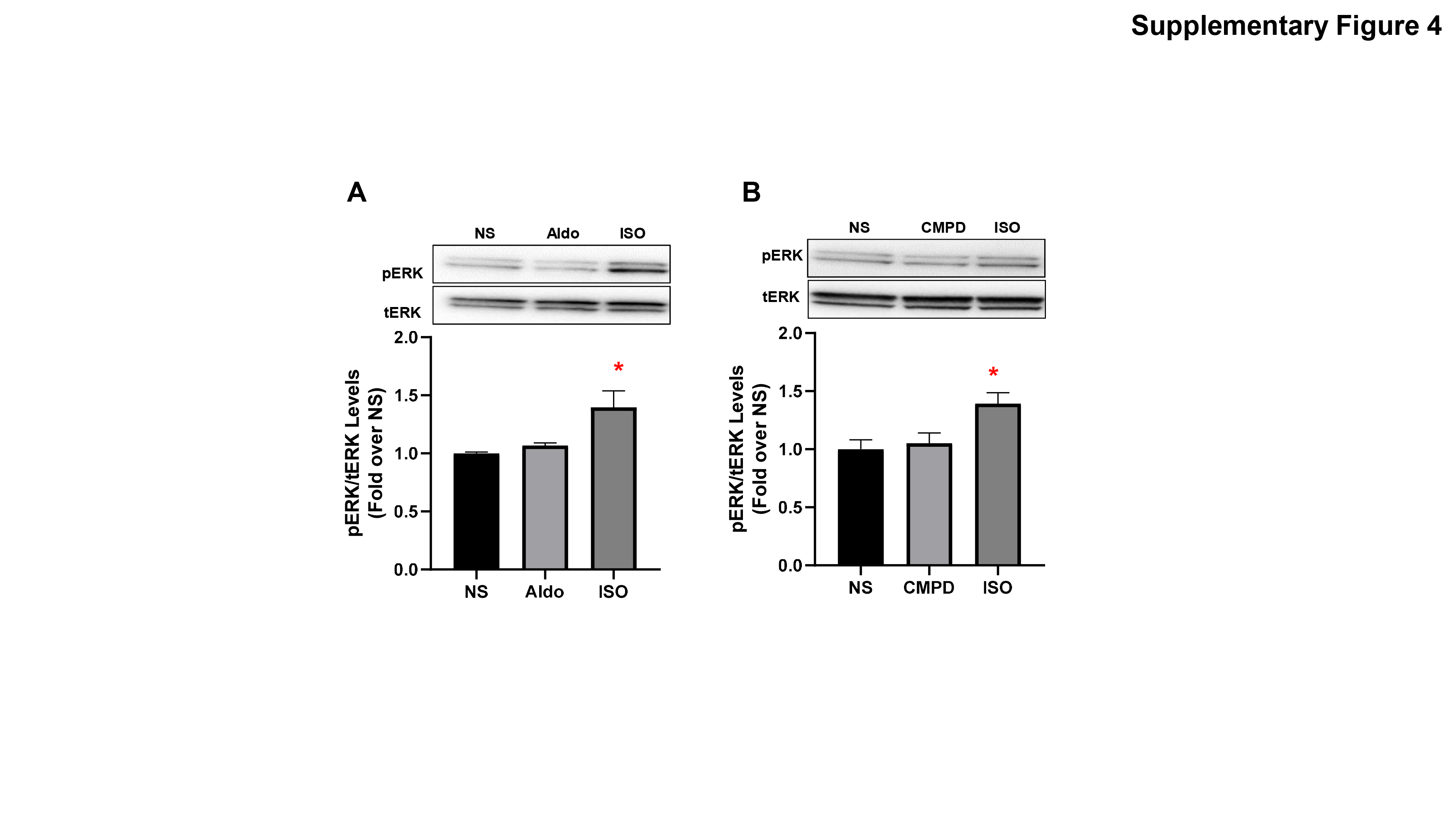

Supplement: Supplementary file 6 [file Image_4.tif]

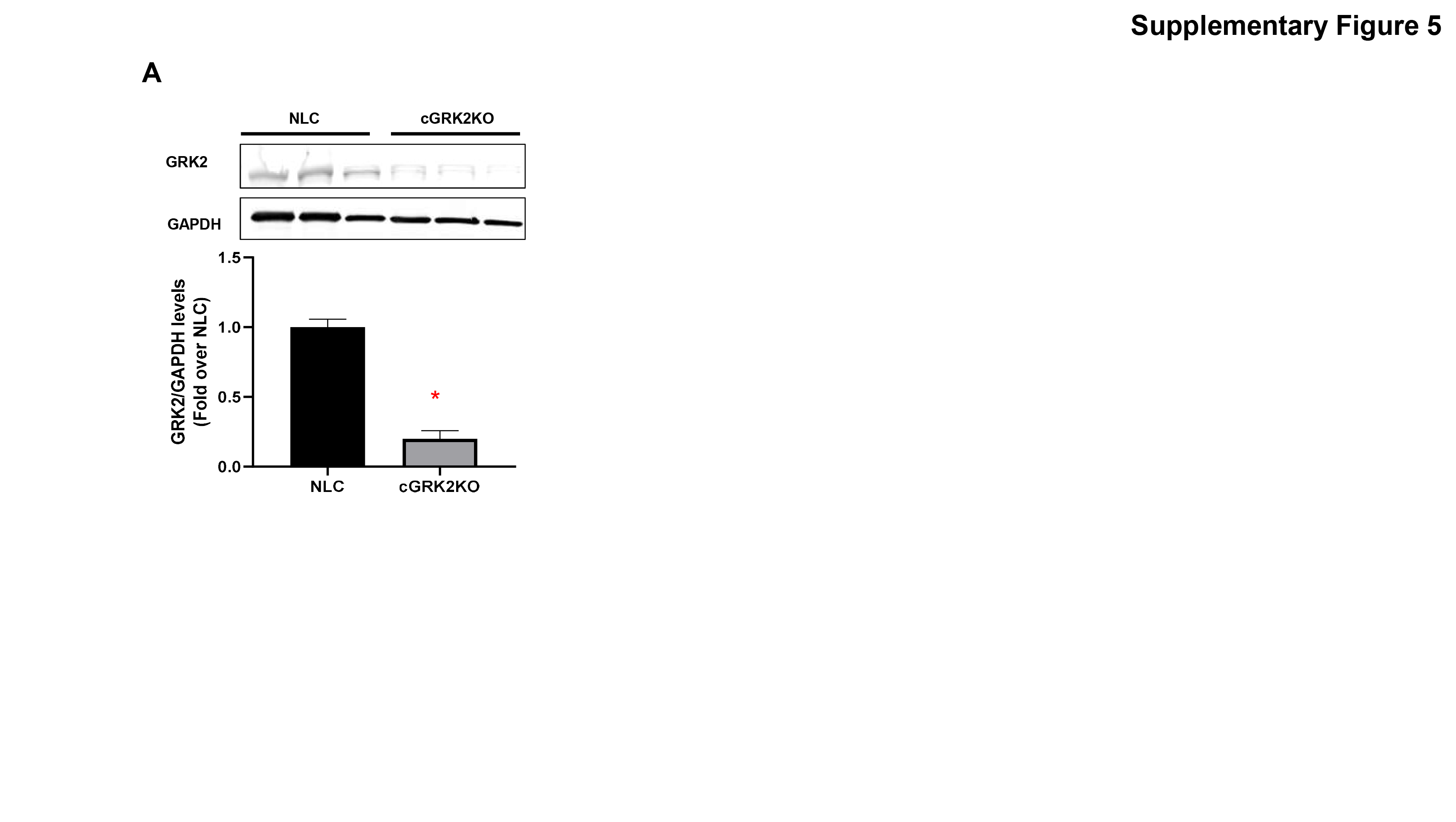

Supplement: Supplementary file 7 [file Image_5.tif]
